# A Smart Band for Automatic Supervision of Restrained Patients in a Hospital Environment

**DOI:** 10.3390/s20185211

**Published:** 2020-09-12

**Authors:** Rubén Muñiz, Juan Díaz, Juan A. Martínez, Fernando Nuño, Julio Bobes, Mᵃ Paz García-Portilla, Pilar A. Sáiz

**Affiliations:** 1 Department of Computer Science, University of Oviedo, 33203 Gijón, Spain; 2 Department of Electric, Electronic, Computer and Systems Engineering, University of Oviedo, 33203 Gijón, Spain; jdiazg@uniovi.es (J.D.); jamartinez@uniovi.es (J.A.M.); fnuno@uniovi.es (F.N.); 3 Department of Psychiatry, University of Oviedo, 33006 Oviedo, Spain; bobes@uniovi.es (J.B.); albert@uniovi.es (M.P.G.-P.); frank@uniovi.es (P.A.S.)

**Keywords:** mechanical contention, monitoring, telemonitoring, telemedicine, real-time, vital constants, sensors, data acquisition, WiFi, BLE, IoMT

## Abstract

Mechanical contention (MC) is a restrictive, vital but controversial measure, prescribed in the majority of EU countries to handle patients with psycho-motor agitation that do not respond to other types of intervention, with an imminent risk of physical violence and aggression involved. This last resort approach implies risks for the somatic health of the contained individual that go from trauma injuries to, in some extreme cases, sudden death. Despite these risks, somatic supervision and the monitoring of patients under MC is limited, being periodically and manually carried out by nursing personnel with portable equipment. In this context, ensuring continuous monitoring using fully automated equipment is an uncovered yet urgent need. There are several devices already in the market capable of monitoring vital signs, but they are not specifically designed for these type of patients and they can be expensive and/or difficult to integrate with other systems from a software perspective. The work described in this paper gives answers to these necessities with the introduction of a low-cost system, targeted at psychiatric patients, for the acquisition and wireless transmission in real-time of physiological parameters, making use of micro-controllers for collecting and processing sensor data, and WiFi technology to upload the information to the server where a patient’s profile with all the relevant vital parameters resides. In addition to data collection and processing, an application aimed at use by nursing staff has also been developed to raise alerts in case any critical condition is detected.

## 1. Introduction

**Figure 1:**
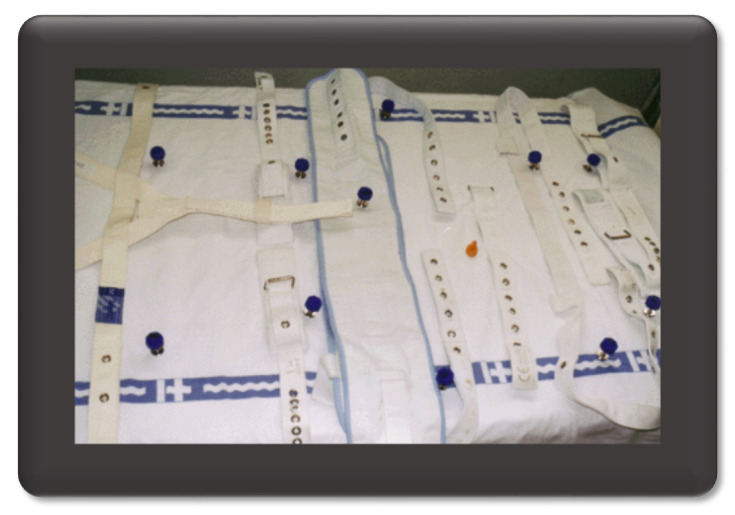
Typical mechanical contention (MC) equipment, based on different belts.

As opposed to other fields, the European Union lacks an agreement in the definition of standards for quality clinical practices in mental health care. This lack of consensus is especially relevant in the case of mechanical contention (MC) as the empirical evidence about its effectivity and safety is limited, in addition to the fact that Article 3 of the International Law on Human Rights states that no human being can be the object of torture or inhuman punishment/degrading treatment. Mechanical contention is a restrictive, vital but controversial measure, prescribed in the majority of EU countries to handle patients with psycho-motor agitation that do not respond to other interventions, with an imminent risk of physical violence and aggression involved. It is an intervention where, using devices approved by the regulatory agencies, normal physical activity is prevented to ensure the safety of a patient and his/her environment. In Figure 1 the belt equipment used is shown, which is always done so under strict regulations. This measure is used to a greater or lesser extent and within a variable period of time in the Acute Psychiatric Unit (APU). Nevertheless, it must be emphasised that MC is also used on patients suffering from severe psychomotor agitation in the other units of hospitals (geriatrics, internal medicine, etc.) along with people treated by other care services.

Besides the aforementioned issues, it is also true that MC can cause major problems, even death [1]; the nursing staff shortage makes continuous patient monitoring impossible, which leads to situations of risk. The patients can suffer seizures and the mechanical restraint can exacerbate them within a short time period. Therefore, it is critical to have a fast response to these seizures, especially in these kinds of patients.

Since these patients can not be monitored 24/7 by the personnel, this work proposes a monitoring system to perform continuous supervision and alert the nursing staff when a complication appears. This will ensure that an adequate clinical response will be provided, preventing a potential life-threatening situation. The proposed system relies on the use of commercially available sensors and a well-known and inexpensive micro-controller, that provides many connectivity options along with wireless capability. This allows the resulting prototype to communicate with a cloud-based database server for storage purposes and send alarms directly to a mobile device via push notifications.

This paper is structured as follows: first it is necessary to determine the set of parameters to measure and store. This decision is based on the observation of psychiatric patients in combination with clinical criteria. Afterwards, the hardware design of a prototype will be discussed, with special emphasis on the target patients in combination with other criteria such as: cost, size, connectivity and upgrade options. This work shows the more relevant aspects of the hardware and software design and finishes with the introduction of some of the obtained results during the tests, that validate the proposed equipment: a low cost wearable device, with enough accuracy to collect data suitable to continuously monitor patients under mechanical contention.

## 2. System Specifications

To be able to provide a quick response when needed, it is necessary to collect and supervise a number of vital signs, such as:

- Blood pressure.
- Body temperature.
- Humidity. When a patient gets nervous or extremely agitated, sweat will appear.
- A rise in the heart rate (HR), measured in beats per minute (BPM), is also a sign of an abnormal situation.
- Breathing rate.
- Sudden movements (acceleration changes) also provide valuable data to detect behavioural changes in a patient.

In order to select the right sensors to measure the aforementioned parameters, it is essential to consider that they are going to be fitted in close contact with the human body. Due to the nature of the patients, it is highly important to make the whole device comfortable [2] and difficult to remove. These constraints narrow the spectrum of sensors and micro-controllers suitable for this device, as size and power consumption will be limiting factors to be considered.

The first step to take to reduce the size as much as possible will be to determine the minimum set of sensors needed. For this reason, blood pressure and breathing rate must be discarded as they can not be precisely measured comfortably. Conversely, body humidity, temperature, acceleration and heart rate can be included as they comply with the imposed size and comfort restrictions. Even though skin contact will be required to measure the heart rate, body temperature and humidity, that will be easily accomplished if all the sensors are fitted in a small device, like the one found in chest straps typically used in sports. It is also interesting to point out that heart rate can be measured using different approaches, but those which rely on the analysis of an electrocardiogram (ECG) are the most accurate ones. In fact, heart rate monitors (HRMs) like those present in the aforementioned chest straps always rely on the ECG approach as it is not an area suitable for the use of optical sensors, commonly found in wearables (activity bands, smartwatches, etc.). With regards to sudden movement detection (acceleration), physical contact will not be required as that information will be provided through a common Inertial Measurement Unit (IMU).

With regards to the control unit and keeping in mind the size and power restrictions, it is vital to find a micro-controller with a small footprint and embedded wireless connectivity, such as Bluetooth, WiFi, Lora, Zigbee, LoRa, LoRaWan and specific radio-frequency handshakes [3], and even 4G/LTE technologies [4,5]. The final selection will be carried out considering different issues, such as:

- **Power consumption.** The communications and control stages are typically the ones with the biggest energy bill. It is essential for this kind of system to minimise this aspect.
- **Size and volume.** Since the equipment is going to be wearable the final size should be kept as small as a possible.
- **User friendly.** The equipment is going to be used by people not necessarily trained in electronic or telecommunications technologies, therefore the developed system has to be easy to configure, and accessible from widely used devices by the general public, such as smartphones or tablets.
- **Cost.** The cost of the circuitry is always a relevant factor, but not necessarily in this case given the field of application of the device.

From the previously introduced system specifications, it can be inferred that there are a number of alternatives available to build the final device. The full design (hardware and software) will be conveniently discussed in the next section.

## 3. Hardware Design and Implementation

**Figure 2:**
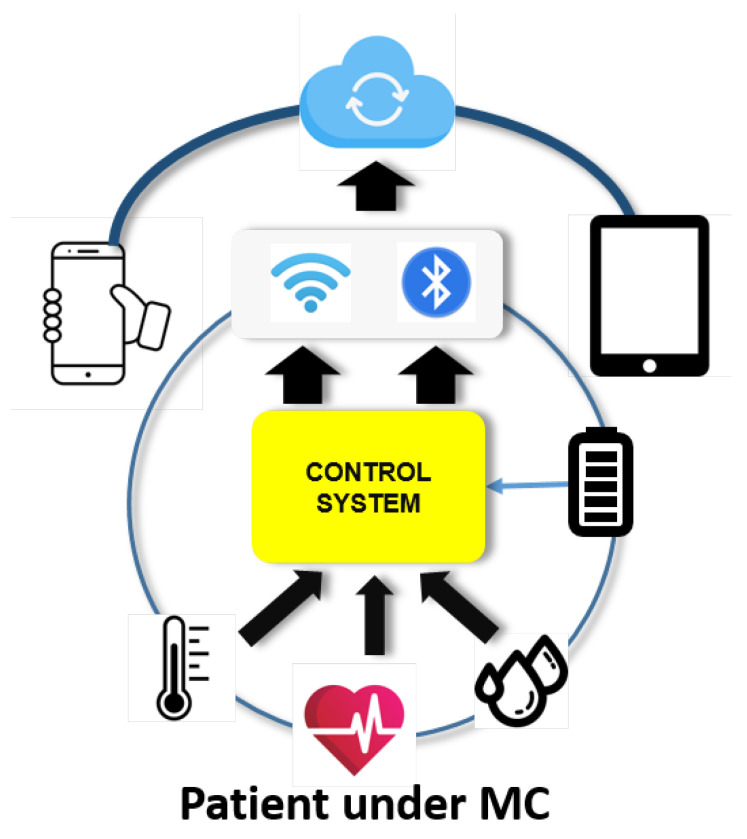
Block diagram for the proposed system: the patient’s sensor data are uploaded to the cloud continuously.

Bearing in mind the aforementioned specifications, the proposed diagram block is depicted in Figure 2. Besides the data upload to the cloud, the equipment should be able to detect early signs of seizure and send alarms, and even identify them before they happen by recognising deviations from normal activity patterns [6]. As the system is based on a controller, there are many options for the implementation, grouped as follows:

- **Complete computer systems based on a System on Chip (SoC).** Raspberry Pi and its clones, Beagle Bone, etc.
- **Micro-controllers.** Arduino, ESP8266/32, etc.

All the aforementioned controllers are capable of performing the required tasks: sensor data collection, analysis, cloud upload, and alarm raising, but the choice must be carried out in terms of size and power consumption.

**Figure 3:**
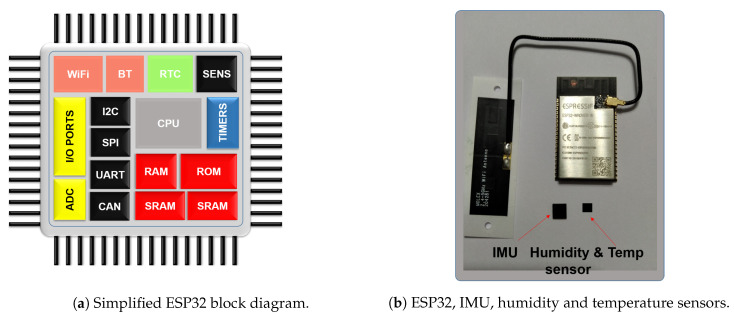
Hardware overview.

There are a number of alternatives already on the market to comply with these requirements, but the well-known ESP32 from Espressif positions itself as the sensible choice, as it has embedded wireless connectivity: WiFi and Bluetooth, along with other useful blocks [7]. This micro-controller has been used in different applications, including medical instrumentation devices [8,9]. The system is completed with temperature, humidity, movement and HR sensors. Figure 3 shows some of the components and a simplified ESP32 block diagram.

The selected sensors include the SHT21 for humidity and temperature, and the MPU-9250 for the inertial unit. As far as heart rate monitoring is concerned, the AD8232 circuit is used. Besides the small size of the aforementioned components, they all use serial communications to interface with the ESP32, simplifying the connections and ensuring adequate data transfer with negligible noise.

**Figure 4:**
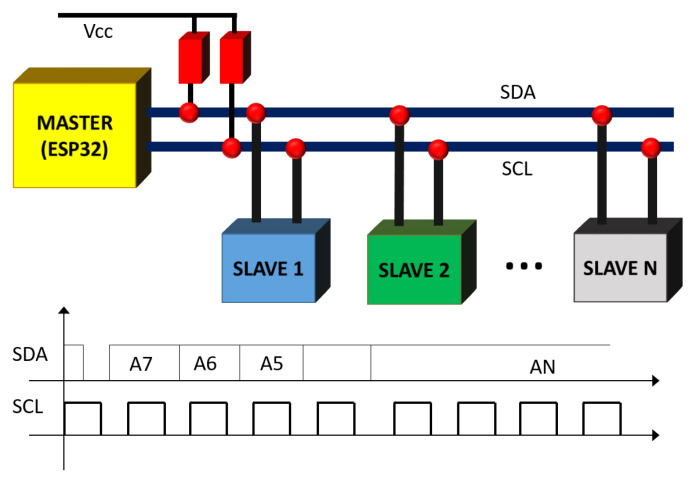
A typical $I^{2}C$ chain and waveforms: one master and several slaves. The connection hardware needed is reduced to two pull-up resistors.

Typically, these sensors rely on either the standard Inter-integrated Circuit ($I^{2}C$) or the Serial Peripheral Interface (SPI) to transmit data to and from a microprocessor (sometimes they might implement both). For the purpose of building the proposed system, any one of the alternatives is valid as both offer sufficient transmission speed. In addition, by using a master-slave architecture they offer the possibility of connecting many devices to the same line. Figure 4 shows a typical ($I^{2}C$) communication system and its main waveforms: all the slaves receive the message, but only one will process it. The slave is selected by the controlling hardware. A complete description of both interfaces can be found in [10,11,12].

The final choice will be determined by the interface exposed by the sensor’s integrated circuit (IC). In this case, both the SHT21 and the MPU-9250 use an $I^{2}C$ bus. The AD8232 outputs an analogue signal that represents the ECG of a heart, therefore it will be connected to one of the A/D pins of the ESP32.

With regards to the power supply, a 3.7 V, 2.6 Ah, 9.62 Wh battery was selected for the prototype. The overall power consumption is basically determined by the WiFi connectivity. When it is in use, the current flow can reach several amperes for a short period of time. When doing other tasks, such as sensor data reading, the measured current barely gets to 100 mA. Consequently, the average current used by the system can be approximately calculated to be as low as 100 mA, thus enabling a single battery charge to last 24 h. However, with the actual current shape it is expected that the battery efficiency will be reduced, implying that the total operation time will be equally affected. Bearing this situation in mind, a low-battery level alarm has been included in the system to alert the nursing staff when the battery reaches such critical levels.

## 4. Communications and Software Design

In the previous sections, it has been stated that the purpose of the device is to measure different bio-signals and upload them to a remote server for further analysis. As the ESP32 device is a micro-controller (not a fully equipped computer system, such as the Raspberry Pi), direct communication with a remote database cannot be established. Even if that possibility were available, it is not recommended for different reasons, such as security and database management system (DBMS) independence. A typical way to overcome that limitation is to use some previously implemented Web services that provide an additional layer between the DBMS and the application. This also enables the participation of additional devices running different operating systems, such as smartphones, tablets, etc.

Nowadays, there are a number of frameworks already available for Internet of Things (IoT) projects, some of them free but limited in functionality. These frameworks provide Web services to allow IoT enthusiasts and professionals to upload sensor data in order for it to be visualised on different devices, such as Android smartphones.

Another approach would be to set up a server (in-house or cloud-based) with a set of custom made Web services along with a DBMS to store the collected sensor data. For this project this option was the preferred one given the sensitive nature of the information (health data) and the required post-processing to detect behavourial anomalies in hospitalised patients. For this purpose, a simple RESTful API using Python and Flask was implemented in combination with a MySQL DBMS. Data were properly encrypted using HTTPS to communicate with the ESP32 and the server.

### 4.1. Sensor Data Acquisition

As this development is focused on psychiatric patients, the minimum set of sensors required to detected seizures are listed as follows:

- Temperature;
- Humidity;
- Heart rate;
- Acceleration in three axes.

During the preliminary tests it was determined that when a patient is severely agitated perspiration (measured as body humidity) increases, along with heart rate and the acceleration measured by the IMU. Temperature is not a critical parameter for this purpose, but the sensor comes in the same package as the humidity one and it is always a standard parameter regularly checked by the nursing staff.

**Figure 5:**
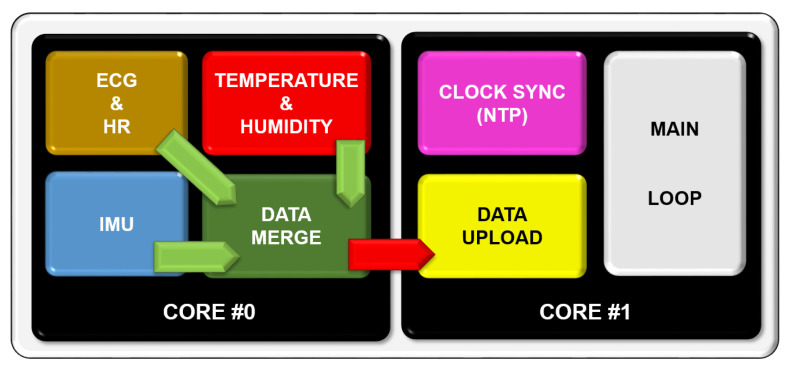
Task definition and message queues. One core is used for data acquisition and processing while the other one is left for communication tasks.

As the ESP32 micro-controller provides two cores, accessible through a FreeRTOS [13] implementation, data acquisition has been split up into different tasks and assigned to one of the cores. As can be seen in Figure 5, **core #0** contains four tasks in total assigned to different sensors: temperature and humidity (determined by the sensor choice described in the next chapter), IMU (Inertial Measurement Unit), ECG (Electrocardiogram) and HR (Heart Rate). The fourth task receives data from the other three and packs them properly before uploading. This data transfer is carried out by means of the message queues available in FreeRTOS.

The message structure for every sensor task includes, apart from the read values, a timestamp which will be used to combine the information from all sensors. This extra field is necessary as the sampling rate might be different. The IMU, for instance, will require more temporal resolution as its values will change more frequently than the others. For this reason, sensor data that is acquired less frequently will need to be repeated in the combined structure sent to the data upload task. During the initial tests it was determined that the sampling rate for the accelerometer should be at least 10 Hz. This value was determined after direct observation of patients. As the device is meant to be attached to the upper part of the body, it is unlikely for a person to be able to move the torso more than five times per second. For the other values it is enough to acquire them once per second as they will not change so quickly.

With those sampling rates in mind, the message payload will be composed of six floating point values. As each datum will be represented using 32 bits, the total size of a sensor data structure will be 24 bytes (6 floating point values). Conversely, the final data rate will be 240 bytes/s as these messages will be uploaded to the server 10 times per second.

### 4.2. ECG Processing and HR Calculation

**Figure 6:**
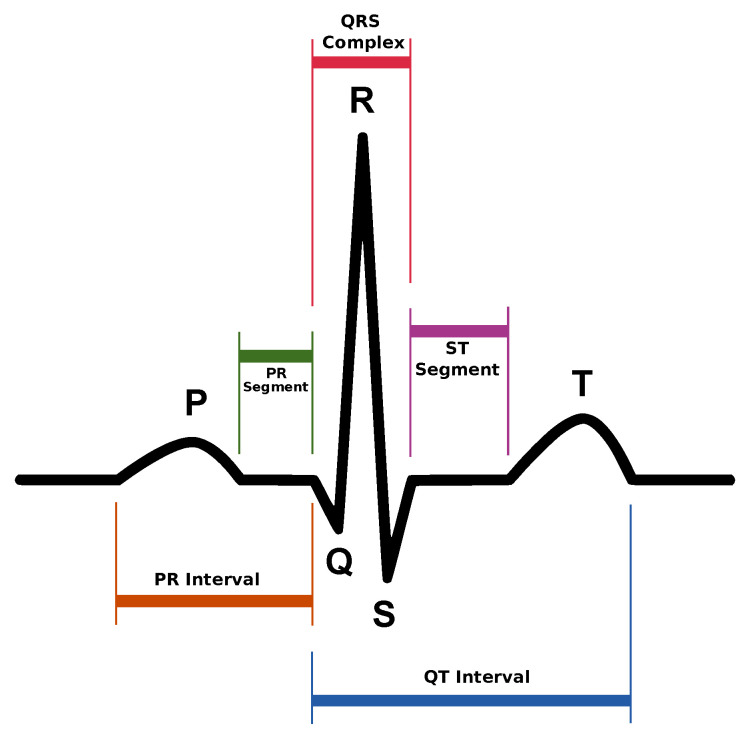
Graphical representation of a heart beat as found in an ECG (created by [15]).

An electrocardiogram (ECG) [14] is a graphical representation of the activity of the heart. A single beat (see Figure 6) is composed of several sections enumerated as follows:

1. P wave;
2. PR interval;
3. PR segment;
4. Q wave;
5. QRS complex;
6. R wave;
7. S wave;
8. ST segment;
9. QT interval;
10. T wave.

These components found in a heart beat contain valuable information in order to diagnose different conditions of the human heart. Nevertheless, as the goal of this work is to simply calculate the heart rate (HR), expressed in beats per minute, the only required parameter to take into account is the R wave, which is the strongest one and appears as a result of ventricular contraction. Consequently, the elapsed time between two successive R waves (contractions) is the only parameter needed to calculate the heart rate, according to Equation (1), where $TR_{1}$ and $TR_{0}$ represent the instant in time (in milliseconds) when two R waves are detected.

(1) $$HR=\frac{1000}{TR_{1}-TR_{0}}\times 60$$

### 4.3. Data Upload

In the introduction of this section the chosen approach was described to communicate the sensor band with the database server. Using a custom Web service, the device can upload the sensor data to the DBMS using the standard HTTPS protocol and JSON to provide a standard representation.

As seen in Figure 5, the data upload task is assigned to **core #1**, along with an additional task to synchronise the ESP32’s internal clock with an NTP server and the main loop. This core is also responsible for the Bluetooth and Wi-Fi stack, thus making it more suitable to contain all the tasks that need to perform some sort of communication.

As is the case of all communication protocols, it is not efficient to send small packets of data because of the overhead introduced by the session opening. For this reason it was determined to be more efficient to send several measurements at once to maximise communication effectiveness. This is accomplished by sending the keep-alive header (invoking the *setReuse(true)* method of the Arduino library).

### 4.4. Seizure Detection and Alarm Sending

The final purpose of this device is to alert the nursing staff when an unwanted situation (severe agitation) is detected. These events take the form of a push notification sent to a mobile device. For this purpose the Firebase Cloud Messaging [16] was selected as it works on multiple platforms, including the ESP32 micro-controller.

The seizure detection can be implemented locally and/or remotely, depending on the complexity of the approach followed to do so. In the first case, historical data from the different sensors will be kept internally in the device and sudden variations during that time window will be detected. When any of the parameters exceed a predefined value, an alarm will be sent to a simple Android application that has been implemented to test this feature. That event will include information about the device’s ID and the parameter out of range. It has also been included a special alarm to warn the nursing staff when a device is running out of battery.

When a patient becomes agitated, heart rate and perspiration will begin to rise and at some point they will increase above a certain threshold. In this case no additional preprocessing is needed. Data from the accelerometer data will require more computation as it contains information from three axes and a gravity component. In essence, the desired value to be studied will be the module of the acceleration as sudden movements of the patient will be totally random. Equation (2) shows the full expression to calculate it from the three components provided by the IMU (aX,aY,aZ), in g-units. It also needs to be pointed out the fact that this calculation is only used to implement the local seizure detector. As was mentioned in Section 4.1, the raw readings provided by the IMU are still uploaded to the cloud server to allow further analysis of the data in a future work.

(2) $$\|\vec{a}\|=\sqrt{aX^{2}+aY^{2}+aZ^{2}}$$

When a patient is in idle state, values obtained using Equation (2) will be close to 1 (due to gravity component present in the IMU readings), as opposed to those calculated during a seizure.

Finally, in order to implement this simple detector, a boolean combination of the parameters involved will take place. This is a key point as, for instance, a sudden variation of the humidity value is not enough, nor violent movements alone as detected by the IMU.

This approach is the one that has been selected for this work as it is simple to implement and valid for testing purposes. The remote alternative constitutes a more refined one as it relies on the additional storage and processing power offered by the server. In fact, a more complex detection scheme can be implemented using machine learning techniques, but this requires more data available from several patients and it is beyond the scope of this work. The first step on this direction has been taken as data are already being uploaded to the cloud for future developments.

## 5. Developed Prototype and Experimental Results

**Figure 7:**
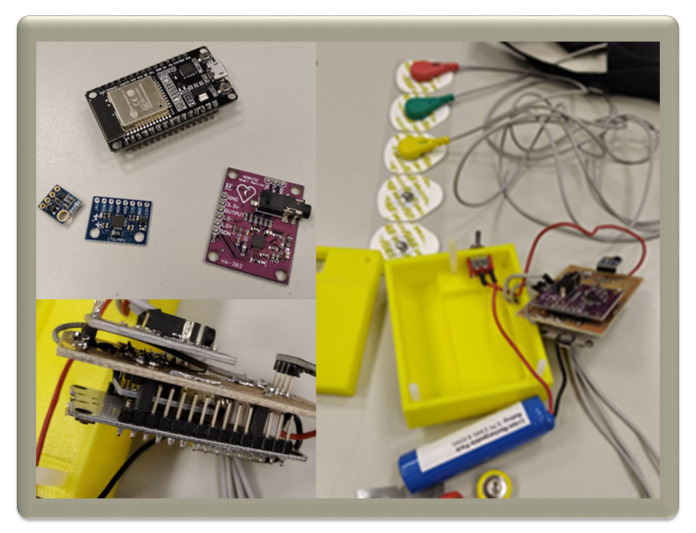
Some prototype pictures. It was tested on some of the authors under mechanical contention, following a protocol designed by psychiatric specialists.

In order to verify the aforementioned system, a prototype was built and tested on four of the authors, following a protocol designed by psychiatric specialists to simulate the response of real patients under mechanical contention. The breadboard is shown in Figure 7. This working prototype is not optimised in terms of size as the final equipment is intended to be attached to a belt or strip, in direct contact with a patient’s body.

The elements that have been used for this initial prototype are:

- ESP32, which comprises the CPU, input/output ports and communications.
- MPU-9250. A very cheap IMU. As was previously mentioned, the main goal is to detect when a patient is having a seizure. Accelerometer data will be extremely useful to detect sudden movements.
- SHT21. Temperature and humidity sensor, in order to detect if the patient is sweating and/or getting a temperature.
- Battery. The battery capacity ensures a period of at least eight hours; as was previously mentioned, the time is unpredictable, but in any case, a low battery-level alarm has been implemented.
- AD8232. Device that provides an analogue signal that represents an ECG. The heart rate can be easily obtained from it.

**Figure 8:**
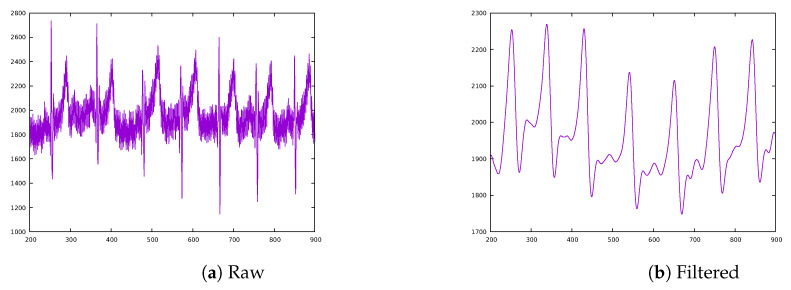
Raw vs. filtered ECGs from the AD8232.

It should be noted that all the sensors can be replaced if more accuracy is needed, but data obtained as a result of the tests was good enough to fulfil expectations. On that thought it is important to note the fact that the AD8232, as opposed to other ICs available on the market, does not provide the HR value directly. It outputs an analogue signal that represents the ECG of the patient’s heart along with a huge amount of noise (Figure 8a), therefore some processing is needed to get a clearer representation. For this prototype a simplification of the Pan–Tompkins algorithm was used [17]. First, a Butterworth filter was used, allowing the signal to look closer to a real ECG (Figure 8b). From this filtered signal, a simple threshold is applied to isolate the R peaks and convert it into a binary stream. Those peaks will be then represented by 1s, thus simplifying the process of measuring the elapsed time between two successive R waves.

**Figure 9:**
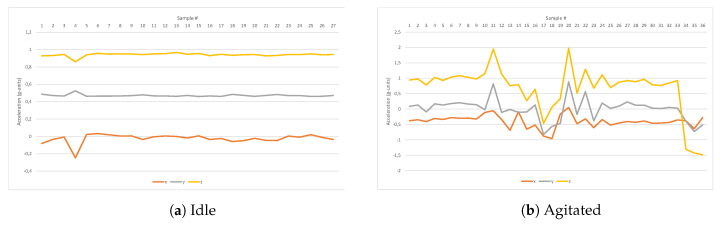
Acceleration data of an idle vs. agitated patient.

**Figure 10:**
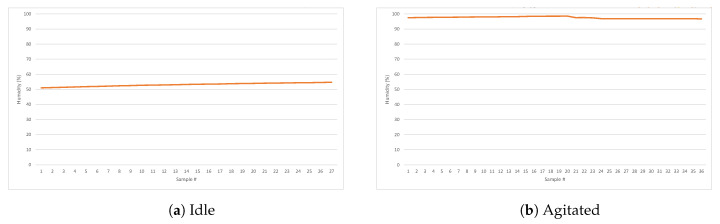
Humidity (perspiration) of an idle vs. agitated patient.

As was previously mentioned, the agitation state involves a number of sudden and violent movements that are precisely captured by an accelerometer. A graphical representation of the data obtained from the IMU for an idle and an agitated patient can be seen in Figure 9a,b, respectively. From those signal representations it seems obvious to establish that the information provided by the accelerometer will be vital to detect changes in a patient’s status, especially when combined with the other measured magnitudes. It is also relevant the huge variations observed in perspiration, as shown in Figure 10.

The system described so far can be expanded with more sensors, whatever the handshake they use: analogue, I2C, SPI as the control unit used is powerful enough to handle more. However, the tests carried out envisage that it is not necessary to include more data to successfully detect a seizure. Nevertheless, keeping in mind that this device is aimed at psychiatric patients, this possibility is left open to fulfil the needs of other types of individuals, such as people suffering from COVID-19 with acute breathing problems. For this specific challenge, it is possible to include an oxygen saturation sensor, and raise an specific alert when the value falls below a preset threshold.

In addition, the device can be easily adapted to comply with HL7 standards [18] in order to exchange data in a hospital environment.

## 6. Conclusions

In private and public health systems, one of the highest costs involved is that of nursing staff. Most of the time, health workers are completely overwhelmed with lots of patients to look after. The proposed system tries to ease nursing tasks by providing a complete, low-cost, flexible and fully-automated monitoring system. The prototype built for this work measures different vital signs (pulse, temperature, etc), and is able to analyse the collected data, upload them to the cloud for in-depth analysis and send alarms to the staff to allow them to take the required actions when necessary. As it was custom-made for the needs of psychiatric patients, this device will be extremely useful to prevent deaths as a result of mechanical contention. It needs to be remarked that even though there are several devices in the market capable of monitoring vital signs (smartwatches, for instance), they are not specifically designed to be fitted to these type of patients, their cost might be high and they lack the required sensors.

From an economic point of view, the total cost of the prototype is very low due to the reduced cost of the chosen components. This will allow a future commercial device to be affordable, given the usually high prices linked to medical equipment.

The application area of the developed system is not limited to psychiatric patients under mechanical contention, as it can be enhanced with more sensors to be used with people suffering from other conditions that also require continuous monitoring. That is nowadays the case with COVID-19 patients as health care professionals have to face risks when they enter a room with highly contagious individuals.
